# Switching Vedolizumab from IV to SC Injection in Inflammatory Bowel Disease Patients with Active Disease: Real-World Experience from a German IBD Cohort

**DOI:** 10.3390/jcm12247657

**Published:** 2023-12-13

**Authors:** Alica Kubesch, Nina Kruse, Florian Jungheim, Ümniye Balaban, Katharina Stratmann, Kathrin Sprinzl, Antje Dienethal, Thomas Krause, Stefan Zeuzem, Irina Blumenstein

**Affiliations:** 1 Goethe University Frankfurt, University Hospital, Medical Clinic 1, 60596 Frankfurt a.M., Germany; kubesch@med.uni-frankfurt.de (A.K.); n.kruse@mail.web.de (N.K.); f.jungheim@med.uni-frankfurt.der (F.J.); k.stratmann@med.uni-frankfurt.de (K.S.); dienethal@med.uni-frankfurt.de (A.D.); zeuzem@em.uni-frankfurt.de (S.Z.); 2 Institute of Biostatistics and Mathematical Modeling, University Hospital, Goethe University, 60596 Frankfurt a.M., Germany; balaban@med.uni-frankfurt.de; 3 Gastroenterologie Opernstrasse, 34117 Kassel, Germany; krause@go-ks.de

**Keywords:** IBD, Crohn’s disease, ulcerative colitis, biologics, real-world data, vedolizumab, sub-cutaneous application, trough levels

## Abstract

Background: Vedolizumab (VDZ) for subcutaneous (SC) injection was approved for use in Europe in 2020 and the US in 2023. Promising efficacy and tolerability have been proven in pivotal trials. However, real-world data on the SC use of VDZ, especially in patients with active disease, are still lacking. We aimed to determine treatment persistence and the drug’s efficacy in inflammatory bowel disease (IBD) patients with active disease in comparison to patients in clinical remission. Methods: Patients treated for IBD in a tertiary care center from July 2020 to December 2021 were included in this study. Clinical and biochemical parameters and data on treatment adherence were collected. VDZ trough levels and disease activity before and after the switch from intravenous (IV) to SC injections were monitored during routine checkups and were retrospectively analyzed. The patients were followed up until week 20. Results: Eighty-two patients were included in the study. Of them, 35 patients had active disease (35/82 = 43%) at the time of the switch and 47 patients (47/82 = 57%) were in remission. In total, 10 patients experienced switch failure, 5 were switched back to IV VDZ, and 5 were swapped to a different biologic agent. We observed an increase in VDZ trough levels from the switch to week 8 and from the switch to week 20 in the remission group. Vedolizumab trough levels of 7.4, 51.4, and 33.45 ug/mL at the switch, week 8, and week 20 were identified to discriminate between remission and disease activity in our cohort. There was no new safety signal detected during the study period. Conclusions: The switch from IV to SC VDZ proved to be efficient, safe, and even capable of reducing residual disease activity.

## 1. Introduction

Inflammatory bowel diseases (IBDs, i.e., ulcerative colitis (UC), Crohn’s disease (CD), and inflammatory bowel disease—unclassified (IBD-U)) are immune-mediated chronic inflammatory conditions with a potential life-long need for therapy. To date, there is no curative therapy for these diseases, and the therapy available therefore only aims to achieve the highest possible level of remission and avoid secondary damage [1,2]. The STRIDE II criteria provide a consensus-based guideline to aid physicians in defining short- and long-term goals within the IBD treatment regime and critically assess treatment success [3]. In times where (a) causal therapies are still to be explored, (b) approved therapies are limited by number and show a lack of efficacy and a loss of response, and (c) non-existent prognostic parameters hugely impact treatment success, the loss of efficacy of the initiated therapy must be avoided at all costs, and the possibility of regaining a response is of great importance.

For many years, vedolizumab (VDZ) has been an integral part of the drug therapy regime for UC and CD in patients who cannot tolerate or who have not responded to conventional therapy [1,2,4,5]. Vedolizumab is a humanized monoclonal antibody whose primary mode of action is binding to α4 β7 integrins on gut-homing T helper lymphocytes, thereby reducing their migration into gastrointestinal tissues [6]. The VISIBLE 1 and VISIBLE 2 phase-III clinical trials showed that the subcutaneous application (SA) of vedolizumab was as effective as maintenance therapy in patients with moderately to severely active UC and CD who had a clinical response to IV VDZ induction therapy. The primary endpoint was met in both trials, showing that the proportion of patients achieving clinical remission at week 52 was significantly greater with SC vedolizumab versus a placebo [7,8].

In 2020, shortly after the start of the COVID-19 pandemic, the SC formulation of VDZ was approved by the EU [9], promising rapid relief for IBD infusion centers which needed to reduce patient contact and often only allowed scheduled infusions and emergency contact. Regardless of the occurrence of the COVID-19 pandemic, SC-administered medications are a favorable way for patients to maintain a high degree of independence and provide a high level of motivation for therapy adherence due to the ease of application and performance as time-consuming visits to infusion centers are no longer necessary under these conditions. The advantages to healthcare providers of switching patients to SC therapies include potential cost savings and freeing up capacity within infusion centers.

A recently published real-world study (TRAVELESS) showed that conversion from IV to SC VDZ application in patients with clinical and biomarker remission was very successful. Approximately 90% of patients could be switched with even better clinical remission rates and an excellent therapy persistence rate for up to 24 weeks [10]. However, in this study, most patients received IV infusions every eight weeks. Of them, only about 25% received infusions in shortened intervals of 4 or 6 weeks, and all patients were in deep clinical and even biomarker remission.

In this study, we aimed to determine whether switching patients with active disease to subcutaneous VDZ application is efficient with regard to the improvement of disease activity.

## 2. Methods

### 2.1. Study Population

All patients with UC, CD, and IBD-U receiving at least two VDZ infusions (IV induction) were included. All patients were treated at the Goethe University Hospital IBD outpatient clinic and consented to switching to SC VDZ. **Inclusion criteria:** All patients had to be diagnosed with an IBD and had to be ≥18 years. **Exclusion criteria:** No definitive IBD diagnosis, fewer than two IV VDZ infusions, refusal to switch to SC VDZ, and patients under 18 years of age.

### 2.2. Study Design

This is a retrospective observational study of switching IV VDZ to SC VDZ in patients with active IBD as well as those in remission (control group). Patients who were treated at the IBD clinic of the Goethe University Hospital between July 2020 and December 2021 and met the inclusion criteria were selected for formal analysis. The patients were followed up at two time points (week 8 and week 20).

### 2.3. Study Endpoints and Assessments

#### 2.3.1. Efficacy

The **primary endpoint** was a maintained switch status at week 20 in both patient groups (those with active disease and those in remission).

**Secondary endpoints** were the improvement of parameters for clinical and biochemical disease activity at weeks 8 and 20 and the determination of VDZ trough levels associated with clinical and biochemical remission at weeks 8 and 20.

**Clinical disease activity** was assessed by calculating clinical activity scores (Harvey–Bradshaw index (HBI) or partial Mayo score) [11,12]. **Biochemical disease activity** was determined by measuring fecal calprotectin (FC)**. Active disease** was assumed if FC was >250 µg/g and/or HBI was >4 or partial Mayo score was >2. **Remission** was assumed if the clinical and biochemical parameters for disease activity indicated remission (FC below 250 µg/g and HBI ≤4 for CD or a partial Mayo score of ≤2). **Switch failure** was assumed if the patient was switched back to IV VDZ, and **treatment failure** was defined as a switch to another biologic agent.

#### 2.3.2. Safety/Adverse Events

All adverse events (AEs) occurring after the SC VDZ switch were documented. This included their severity, the possible need for concomitant treatment, or changes within the biologic treatment. Adverse events of special interest were serious infections, malignancies, progressive multifocal leukoencephalopathy (PML), liver injury, injection site reactions, general reactions, and hypersensitivities.

#### 2.3.3. Pharmacokinetics and Laboratory Testing

Blood samples were obtained at the switch, week 8, and week 20. At these time points, general laboratory parameters as well as VDZ serum concentrations were measured with enzyme-linked immunosorbent assay (ELISA). All laboratory testing was conducted for routine monitoring.

To assess biochemical disease activity, patients submitted a stool sample (FC) for measurement at the switch, week 8, and week 20.

#### 2.3.4. Statistical Analyses

Statistical analyses were conducted using IBM SPSS Statistics Version 28.0 (International Business Machine Corporation, Endicott, NY, USA) as well as Bias 11.12. *p*-values ≤ 0.05 were considered statistically significant. Numerical data were presented with the median and interquartile range (IQR). To test for differences between CD and UC in metric predictors in terms of age, BMI, and infusions prior to the switch, we employed Mann–Whitney U tests (U). For the categorial predictors of gender, prior biologicals, >1 biologicals, and SC continuation after week 20, we employed Chi² tests (X^2^). If the expected cell frequencies were below 5, we used Fisher’s exact test. The same procedure was used to test for differences between active disease and remission. To test for differences between VDZ trough levels or FC levels between the switch, week 8, and week 20, we employed the Friedman test. To test for differences in clinical and biochemical remission between the switch, week 8, and week 20, we employed Cochran’s Q test. The cut-off value for VDZ trough levels was evaluated by using receiver operating characteristic (ROC) analysis (using patients in remission as negative outcomes). To address the subject of missing values in the dataset, we employed Little’s MCAR test.

### 2.4. Ethics Statement

For this retrospective study, approval from the local Ethics Committee of the University Hospital Frankfurt (No. 2022-708, 21 April 2022) was obtained. This study was conducted in accordance with ethical and data protection regulations.

## 3. Results

### 3.1. Study Population

A total of 82 patients were included in this analysis. In total, **35**/82 = 43% patients were classified as having an **active disease** status, and **47**/82 = 57% patients were classified as being in **remission**. The results show no statistically significant differences in terms of age (U = 789, Z = −0.314, *p* = 0.753), BMI (U = 594, Z = −1.769, *p* = 0.077), or infusions prior to switch (U = 1325, Z = −1.194, *p* = 0.233) as well as gender (X²(1) = 0.083, *p* = 0.773), prior exposure to biologicals (X²(1) = 2.850, *p* = 0.091), the prior number of biological treatments > 1 (X²(1) = 0.536, *p* = 0.464), or SC application continued after week 20 (X²(1) = 1.214, *p* = 0.270), Table 1).

Overall, 50 patients were diagnosed with UC, 26 were diagnosed with CD, and, lastly, 6 were diagnosed with IBD-U. When comparing the patients with UC and CD, the results show no statistically significant differences in terms of age (U = 602.5, Z = −0.520, *p* = 0.603) and BMI (U = 495, Z = −1.461, *p* = 0.144). The number of infusions prior to the switch was significantly lower in the UC group compared to the CD group (U = 466,5, Z = −2.014, *p* = 0.044). Furthermore, we observed significant differences in terms of gender (X²(1) = 4.412, *p* = 0.036, Cramer V = 0.241), prior exposure to biologics (X²(1) = 19.694, *p* < 0.001, Cramer V = 0.516), and the prior number of biological treatments > 1 (X²(1) = 7.033, *p* = 0.008 (Fisher’s exact test), Cramer V = 0.484), but no significant differences in SC application continued after week 20 (X²(1) = 0.040, *p* = 0.842, Cramer V = 0.024). For detailed information by disease entity, please refer to the Supplemental Information (SI) (Tables S1 and S2).

#### 3.1.1. Concomitant Oral Steroids

In total, 11 patients out of 82 (11/82 = 13%) were on oral steroids prior to switching. In addition, 7 of these 11 patients were in the **active disease** group (7/11 = 63.3%). Five patients on steroids and who had disease activity remained on steroids at week 8 (5/35 = 14.3%) and two remained on steroids at week 20 (2/35 = 5.7%).

In the **overall patient population,** six of the initial eleven patients on steroids (6/82 = 7.3%) remained on steroids at week 8 and three patients remained on steroids at week 20 (3/82 = 3.6%). In summary, 96.4% of the patients were taken off steroids at week 20 in comparison to 87% at week 0.

#### 3.1.2. Previous Infusion Intervals or Recent Induction

Of the 35 patients with **active disease** at the switch, 14 patients were on an 8-week interval (14/35 = 40%) and 7 were on a 4-week interval (7/35 = 20%). Six patients in the **remission** group were switched after induction (two infusions of VDZ IV 300 mg, 6/47 = 12.7%), 31 patients were on an 8-week interval (31/47 = 66.0%), and 10 patients were on a 4-week interval (10/47 = 21.3%) (Table 2).

### 3.2. Efficacy

#### 3.2.1. Primary Endpoint: SC Persistence

Eleven patients discontinued SC VDZ treatment up until week 20 (11/82 = 13.4%), corresponding to a SC VDZ persistence rate of 86,6% in the overall cohort.

Switch failure was reported in a total of five patients over the observational period (*5/82 = 6.1%*), all of whom were from the active disease group. Three patients switched back to IV VDZ before week 8 and two patients switched back to IV VDZ between weeks 8 and 20. Of note, one patient from the switch failure group was switched back to IV VDZ due to a local allergic reaction.

A biologic agent change was reported in five (5/82 = 6.1%) patients. One patient underwent surgery due to pre-existing stenosis and was switched to another biologic agent post-surgery before week 8. The remaining four patients were switched to a different biologic agent between weeks 8 and 20. Lastly, one patient from the remission group discontinued SC VDZ as he was in deep remission and elected for treatment discontinuation.

#### 3.2.2. Clinical Remission

For patients with **active disease** at the switch, the results show statistically significant differences (Q(2) = 7.750, *p* = 0.021, *n* = 18). Pairwise comparison post hoc tests were conducted. The results show significant differences between the switch and week 20 (Bonferroni-adj. *p* = 0.028), but not between the switch and week 8 (Bonferroni-adj. *p* = 0.091) or between weeks 8 and 20 (Bonferroni-adj. *p* > 0.999). Thus, significantly more patients were in clinical remission at week 20 when compared to the time of the switch. Because of the inadequate sample distribution, we refrained from repeating the aforementioned analysis for patients with **remission** status.

#### 3.2.3. Biochemical Remission

**Figure 1:**
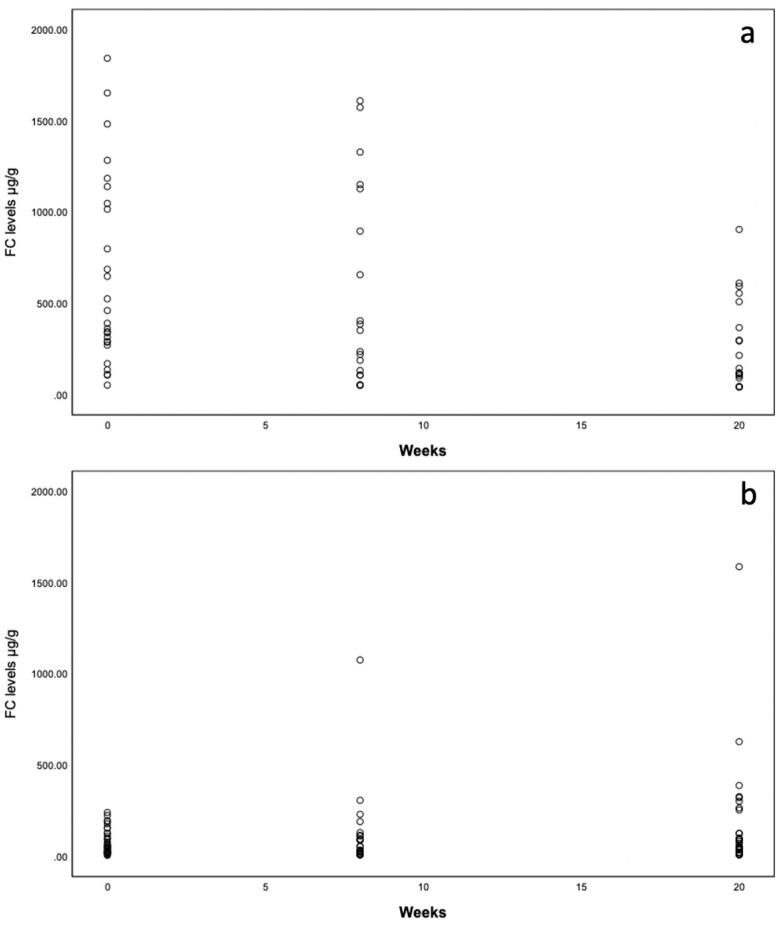
(**a**,**b**) Dot plots of FC levels (µg/g/mL) over time for the switch, week 8, and week 20 for active disease (**a**) and remission (**b**).

For patients with **active disease**, the results show statistically significant differences (Q(2) = 8.857, *p* = 0.012, *n* = 12). Pairwise comparison post hoc tests were conducted. Here, the results showed significant differences between the switch and week 20 (Bonferroni-adj. *p* = 0.016) but not between the switch and week 8 (Bonferroni-adj. *p* = 0.062) or between weeks 8 and 20 (Bonferroni-adj. *p* > 0.999). Therefore, significantly more patients were in biochemical remission at week 20 when compared to the time of the switch. Because of the inadequate sample distribution, we refrained from repeating the aforementioned analysis for patients with **remission** status (Table 3, Figure 1).

#### 3.2.4. Biomarker Efficacy—FC Levels

We did not observe significant differences between the switch, week 8, and week 20 (Chi²(2) = 4.167, *p* = 0.125, *n* = 12) in the **active disease** group or in the **remission group** (Chi²(2) = 5.729, *p* = 0.057, *n* = 15). A trend towards FC reduction from 425 µg/g (median 787.3 µg/g) to 179,5 µg/g (median 420 µg/g) was observed.

Within the remission group, the FC levels remained low at weeks 8 and 20 (Table 4, Figure 1a,b).

#### 3.2.5. Sub-Analysis for the Intensified (i.e., 4-Week) Interval Cohort: FC Level over Time in Relation to Disease Activity at the Switch

Seventeen patients were placed on an intensified schedule, whereby VDZ was given intravenously every four weeks before switching. Seven patients (7/17 = 41.2%) had **active disease** at the time of the switch. Fecal calprotectin levels were compared between patients with **active disease** at the switch and those in **remission**. Data on FC levels are provided in the table below. We observed a reduction in FC levels over time in the active disease group who received IV VDZ in an intensified 4-week interval before switching to SC application (Table 5).

#### 3.2.6. Sub-Analysis for C-Reactive Protein (CRP) Levels by Disease Activity

We measured CRP levels at every time point. Overall CRP levels were low in our cohort. We did not observe significant differences in both groups at the respective time points.

For the **active disease group,** no significant differences in CRP levels were observed between the switch, week 8, and week 20 (X²(2) = 5.017, *p* = 0.81, *n* = 30). Pairwise comparison post hoc tests showed significant differences between the switch and week 8 (Bonferroni-adj. *p* = 0.36) and between the switch and week 20 (Bonferroni-adj. *p* > 0.999) or between weeks 8 and 20 (Bonferroni-adj. *p* > 0.999).

For the **remission group,** significant differences in CRP levels were observed between the switch, week 8, and week 20 (X²(2) = 6.413, *p* = 0.04, *n* = 39). Pairwise comparison post hoc tests showed significant differences between the weeks 8 and 20 (Bonferroni-adj. *p* = 0.045) but not between the switch and week 8 (Bonferroni-adj. *p* > 0.999) between switch and 20 (Bonferroni-adj. *p* = 0.30) (Table S3).

### 3.3. Pharmakokinetics—VDZ Trough Level

For the **entire cohort,** significant differences in VDZ trough levels were observed between the switch, week 8, and week 20 (X²(2) = 39.948, *p* < 0.001, *n* = 39). Pairwise comparison post hoc tests showed significant differences between the switch and week 20 (Bonferroni-adj. *p* < 0.001) and between the switch and week 8 (Bonferroni-adj. *p* < 0.001) but not between weeks 8 and 20 (Bonferroni-adj. *p* > 0.999).

In the **active disease group,** significant differences in VDZ trough levels were observed between the switch, week 8, and week 20 (Chi²(2) = 6.727, *p* = 0.035, *n* = 11). Pairwise comparison post hoc tests showed no significant differences between the switch and week 20 (Bonferroni-adj. *p* = 0.099), between the switch and week 8 (Bonferroni-adj. *p* = 0.057), or between weeks 8 and 20 (Bonferroni-adj. *p* > 0.999).

**Figure 2:**
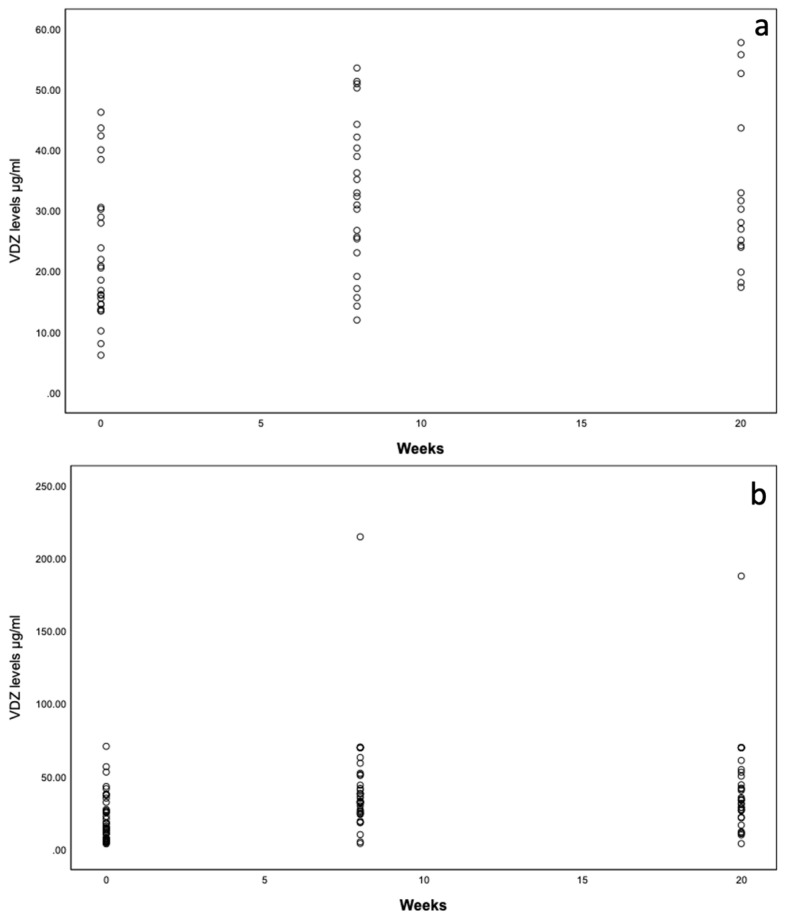
(**a**,**b**) Dot plots of VDZ trough levels (µg/mL) over weeks 0, 8, and 20 for active disease (**a**) and remission (**b**).

In the **remission group**, significant differences in VDZ trough levels were observed between the switch, week 8, and week 20 (Chi²(2) = 34.109, *p* < 0.001, *n* = 28). Pairwise comparison post hoc tests showed significant differences between the switch and week 20 (Bonferroni-adj. *p* < 0.001) and between the switch and week 8 (Bonferroni-adj. *p* < 0.001) but not between weeks 8 and 20 (Bonferroni-adj. *p* = 0.894) (Table 6, Figure 2a,b).

### 3.4. ROC Analysis to Determine the VDZ Serum Concentration Cut-off

**Figure 3:**
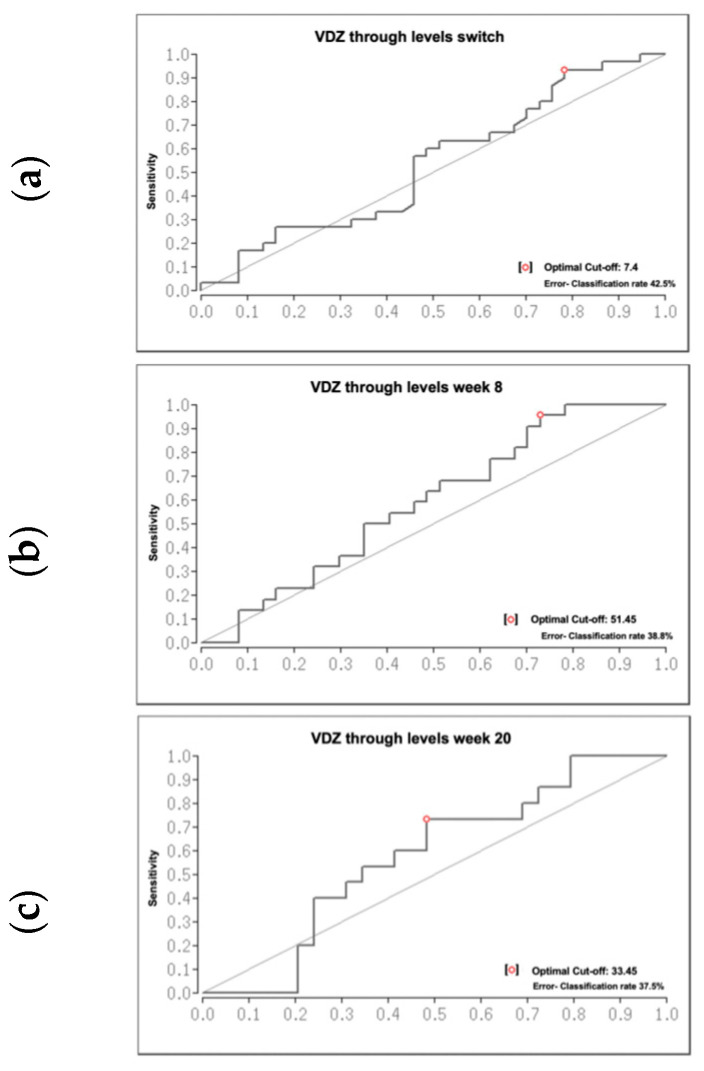
Area under the receiver operating characteristics curve (AUROC) of vedolizumab (VDZ) trough levels. (**a**), VDZ trough levels at switch, (**b**) VDZ trough levels at week 8 and. (**c**) VDZ trough levels at week 20.

The area under the receiver operating characteristic curve (AUROC) was calculated for the entire cohort to determine a cut-off that would discriminate between ***active disease*** and ***remission*** at all three time points. The ROC analysis showed that VDZ trough levels of 7.4, 51.4, and 33.45 µg/mL at the switch, week 8, and week 20 were identified to discriminate between ***remission*** and ***active disease*** in our cohort (Figure 3a–c).

### 3.5. Safety/Adverse Events

Nine patients reported adverse events while receiving SC VDZ (9/82 = 11%). The reported adverse events included acne (*n* = 1), arthralgia (*n* = 1), burning at the injection site (*n* = 1), redness at the injection site (*n* = 1), headaches (*n* = 1), fatigue (*n* = 1), hematoma at the injection site (*n* = 1), night sweats (*n* = 1), and itching at the injection site (*n* = 1). Of the nine patients, two patients stopped SC VDZ due to adverse events. One patient reported severe reddening at the injection site, and a suspected allergic reaction to the solution was suspected. This patient switched back to an intravenous application of VDZ. Another patient switched to an anti-TNF alpha inhibitor due to arthralgia. For all other adverse events, no treatment or concomitant medication was needed. No serious infections, injuries, PML, or malignancies occurred.

### 3.6. Missing Data Analysis

To address the subject of missing values in the dataset, we employed Little’s MCAR test (X^2^(207) = 217.042, *p* = 0.302). We concluded that the missing data points could be ignored in the further analysis.

## 4. Discussion

In this cohort study of 82 patients treated in a tertiary referral center setting, the potential of VDZ treatment optimization by switching from IV to SC formulations was investigated.

In the overall study population, the persistence rate on SC VDZ was 86,6% irrespective of disease status, with 96,4% of patients being taken off steroids at the end of the observation period.

In patients with active disease status, the number of patients in clinical and biochemical remission increased significantly within the **active disease** group until week 20. This improvement was most likely mediated by significant increases in the trough levels at weeks 8 and 20.

Other real-world studies, investigating VDZ switch from IV to SC application have reported similar VDZ discontinuation rates, with them varying from 7,4 to 12.5% [10,13,14,15]. However, in the aforementioned studies, the patients were mostly in deep clinical and biomarker remission as evidenced by clinical disease scores and low FC levels. In the pivotal trials VISIBLE I [7] and II [8], the SC VDZ discontinuation rates were 27.4% (CD) and 38.9% (UC) in the first 52 weeks of treatment. In both trials, the patients were switched to different treatment regimens if they experienced clinical response after two infusions, but they were not necessarily in clinical remission.

Within the **active disease** group in our study, the number of patients in clinical and biochemical remission increased between the switch and week 20. A decrease in fecal calprotectin levels was observed in the **active disease** group. However, when evaluating the FC levels longitudinally, no statistical significance could be observed. Due to the retrospective observational nature of the data included in this study, FC levels were not available for all patients at all time points, therefore limiting the relevance of this analysis. In the **remission** group, no statistically significant changes in biochemical and clinical remission or FC levels were observed, as was to be expected. Other real-world studies on VDZ switching to SC have also reported no significant changes in disease activity for their cohorts when switching in remission [10,13,14,15].

An important aim of our study was the calculation of cut-off VDZ serum levels indicating a successful switch from as early as week 8. The median VDZ concentrations for our entire cohort were 16.9 μg/mL at switch, 33 μg/mL at week 8, and 31.7 μg/mL at week 20. Our results are comparable with the reported concentrations in the VISIBLE trials (34.6 μg/mL for UC and 30.2 μg/mL for CD patients) [7,8] and the study by Volkers et al. [4], who reported VDZ concentrations of 31 μg/mL at week 12 and 37 μg/mL at week 24. We determined VDZ trough levels of 7.4 μg/mL, 51.4 μg/mL, and 33.45 ug/mL at the switch, week 8, and week 20, respectively, to discriminate between **remission** and **active disease** at week 20.

Only nine patients (9/82 = 10.98%) reported adverse events after switching to clinical and biochemical VDZ. Our findings are comparable with other previously published cohorts [7,8,10,13,14,15].

There are several limitations to our study, its retrospective nature being foremost. Data on both clinical and biochemical disease activity were not available for all patients at all time points, thus possibly limiting our observations’ validity. To account for this, a missing data analysis was performed to confirm that the data were missing at random. Another relevant limitation is the lack of a control group, whereby patients with **active disease** at the switch remained on VDZ infusions, especially when they had just completed induction. A recently published study by Lim et al. showed that efficacy and treatment persistence were equally effective for SC applications. However, in the study by Lim et al., mainly patients in remission or with mild disease activity were included [16]. Furthermore, we did not provide endoscopic outcome data, as only a few patients underwent a colonoscopy during the observational period.

Still, our study has its merits as it provides real-world VDZ switch data with a larger proportion of patients with active disease in comparison to previously published studies.

To conclude, switching to SC VDZ was possible and safe in most patients in our cohort, even in those with **active disease**. These results provide an opportunity for VDZ treatment optimization in patients with suboptimally controlled IBD, a finding that is of great importance in times of limited treatment options for IBD patients.

## Figures and Tables

**Table 1:** Patient characteristics by disease activity. The table shows the number of total observations with percentages for the categorical variables and the median with the interquartile range for the metric variables.

	Active Disease	Remission
Age *n*, median (IQR)	*n* = 35, 40 (30)	*n* = 47, 38 (25)
Female gender *n* (%)	*n* = 35, 19 (54.3)	*n* = 47, 24 (51.1)
BMI *n*, median (IQR)	*n* = 33, 23.8 (7.8)	*n* = 47, 26.5 (7.7)
Prior biologicals *n* (%)	*n* = 33, 17 (51.5)	*n* = 46, 15 (32.6)
>1 Biologicals *n* (%)	*n* = 17, 9 (52.9)	*n* = 15, 6 (40)
Infusions prior to the switch *n*, median (IQR)	*n* = 35, 7 (20)	*n* = 47, 10 (13)
SC continued after week 20 *n* (%)	*n* = 29, 19 (65.5)	*n* = 44, 34 (77.3)
Crohn’s disease *n* (%)	*n* = 35, 12 (34.3)	*n* = 47, 14 (29.8)
Concomitant medications *n* (%)	*n* = 35, 18 (51.4)	*n* = 47, 24 (51.1)
5-ASA oral	7	14
5-ASA rectal	1	3
5-ASA oral and rectal	2	2
Sulfasalazine	0	2
Oral steroids at switch	8	3

Body mass index (BMI); subcutaneous (SC).

**Table 2:** VDZ intervals prior to the switch by disease activity. The number of total observations with percentages.

	Active Disease *n* = 35	Remission *n* = 47
Induction, *n* (%)	14 (40.0)	6 (12.7)
8-week interval, *n* (%)	14 (40.0)	31 (66.0)
4-week interval, *n* (%)	7 (20.0)	10 (21.3)

**Table 3:** Clinical and biochemical remission by disease activity. The table shows the total number of observations in the groups and the number of observations with clinical/biochemical remission status. Percentages are shown in parentheses.

	Active Disease *n* = 35	Remission *n* = 47
**Clinical remission**		
Switch, *n* (%)	*n* = 33, 10 (30.3)	*n* = 45, 45 (100)
Week 8, *n* (%)	*n* = 29, 15 (51.7)	*n* = 40, 39 (97.5)
Week 20, *n* (%)	*n* = 18, 12 (66.7)	*n* = 38, 37 (97.4)
**Biochemical remission**		
Switch, *n* (%)	*n* = 28, 5 (17.9)	*n* = 32, 32 (100)
Week 8, *n* (%)	*n* = 19, 9 (47.4)	*n* = 26, 23 (88.5)
Week 20, *n* (%)	*n* = 17, 9 (52.9)	*n* = 32, 24 (75)

**Table 4:** Fecal calprotectin by disease activity. The table shows the number of total observations and the median with the interquartile range.

FC Level	Active Disease	Remission
Switch *n*, median (IQR)	*n* = 26, 426 (787.3)	*n* = 32 58 (99.5)
Week 8 *n*, median (IQR)	*n* = 19, 352 (1020)	*n* = 24, 43 (98)
Week 20 *n*, median (IQR)	*n* = 18, 179.5 (420.5)	*n* = 33, 49 (168.5)

Vedolizumab (VDZ); fecal calprotectin (FC).

**Table 5:** Sub-analysis for the 4-week IV interval cohort at the switch. Fecal calprotectin by disease activity. The table shows the number of total observations and the median with interquartile range.

FC Level	Active Disease	Remission
Switch *n*, median (IQR)	*n* = 4, 406.5 (711)	*n* = 6, 44 (49.8)
Week 8 *n*, median (IQR)	*n* = 4, 81 (265)	*n* = 3, (94)
Week 20 *n*, median (IQR)	*n* = 4, 166 (392.8)	*n* = 6, 46.2 (253.3)

Vedolizumab (VDZ); fecal calprotectin (FCP).

**Table 6:** VDZ trough levels by disease activity. The table shows the number of total observations and the median with the interquartile range.

VDZ Trough Level	Entire Cohort	Active Disease	Remission
Switch *n*, median (IQR)	*n* = 69, 16.9 (18.7)	*n* = 26, 19.6 (16)	*n* = 43, 14.9 (20)
Week 8 *n*, median (IQR)	*n* = 61, 33 (22.15)	*n* = 23, 32.4 (19.1)	*n* = 38, 34.25 (26.15)
Week 20 *n*, median (IQR)	*n* = 48, 31.7 (28.05)	*n* = 15, 28.1 (19.7)	*n* = 33, 33.9 (29.5)

Vedolizumab (VDZ).

## Data Availability

The data presented in this study are available on request from the corresponding author. The data are not publicly available due to data protection and ethical regulations.

## Supplementary Materials

The following supporting information can be downloaded at: https://www.mdpi.com/article/10.3390/jcm12247657/s1, Table S1: Patient characteristics by disease entity. The table shows the number of total observations with percentages for the categorical variables and the median with the interquartile range for the metric variables. Table S2: Disease classification: Crohn’s disease and ulcerative colitis. Table S3: Analysis for CRP levels at switch, week 8, and week 20 by disease activity. The table shows the number of total observations and the median with interquartile range.
