# Supplementary material for: Switching Vedolizumab from IV to SC Injection in Inflammatory Bowel Disease Patients with Active Disease: Real-World Experience from a German IBD Cohort
Source: J Clin Med. 2023 Dec 13;12(24):7657. doi: 10.3390/jcm12247657 (PMC10743496; doi:10.3390/jcm12247657)
Supplement: Supplementary file 1 [file jcm-12-07657-s001.zip › jcm-2674037-supplementary.pdf]

Supplementary Table S1. Patient characteristics by disease entity. The table shows the number of total observations with percentages for the categorical variables and the median with the interquartile range for the metric variables.

	Crohn's Disease	Ulcerative Colitis
Age<br>*n*, median (IQR)	*n* = 26, 37.5 (20)	*n* = 50, 38 (28)
Female gender<br>*n* (%)	*n* = 26, 9 (34.6)	*n* = 50, 30 (60)
BMI<br>*n*, median (IQR)	*n* = 26, 27.7 (5.2)	*n* = 48, 24.1 (8.6)
Prior biologicals *n*, %	*n* = 25, 19 (76)	*n* = 49, 11 (22.4)
> 1 Biologicals *n* (%)	*n* = 19, 13 (68.4)	*n* = 11, 2 (18.2)
Infusions prior to the switch<br>*n*, median (IQR)	*n* = 26, 13.5 (38)	*n* = 50, 7.5 (12)
SC continued after week 20 *n*, (%)	*n* = 25, 19 (76)	*n* = 42, 31 (73.8)

* The six patients with IBD-U are not included in the table. Body mass index (BMI); subcutaneous (SC).

Supplementary Table S2. Disease classification: Crohn's disease and ulcerative colitis.

**Crohn's Disease**	
**Vienna classification**	
**Age at diagnosis**	
A1, *n*(%)	24 (92.31%)
A2, *n*(%)	2 (7.69%)
**Disease location**	
L1 , *n*(%)	7 (26.92)
L2, *n*(%)	5 (19.23)
L3, *n*(%)	10 (38.46)
L4, *n*(%)	1 (3.85)
L1+L4, *n*(%)	2 (7.69)
L3+L4, *n*(%)	1 (3.85)
**Phenotype**	
B1, *n*(%)	9 (34.62)
B2, *n*(%)	3 (11.54)
B3, *n*(%)	9 (34.62)
Unknown, *n*(%)	5 (19.23)
**Ulcerative Colitis**	
**Montreal classification**	
**Disease extent**	
E1	9 (18)
E2	9 (18)
E3	32 (64)
**Disease severity**	
S0	22 (44)
S1	17 (34)
S2	8 (16)
S3	3 (6)

Supplementary Table S3. Analysis for CRP levels at switch, week 8, and week 20 by disease activity. The table shows the number of total observations and the median with interquartile range.

CRP level (mg/dl)	Active Disease	Remission
Switch<br>*n*, median (IQR)	*n* = 32, 0.2 (0.40)	*n* = 44, 0.20 (0.32)
Week 8<br>*n*, median (IQR)	*n* = 31, 0.2 (0.46)	*n* = 42, 0.16 (0.37)
Week 20<br>*n*, median (IQR)	*n* = 30, 0.155 (0.37)	*n* = 44, 0.24 (0.39)

C-reactive protein (CRP).
